# Prevalence of colorectal cancer biomarkers and their impact on clinical outcomes in Riyadh, Saudi Arabia

**DOI:** 10.1371/journal.pone.0249590

**Published:** 2021-05-12

**Authors:** Amjad Alharbi, Haifa Bin Dokhi, Ghadir Almuhaini, Futoon Alomran, Emad Masuadi, Nouf Alomran

**Affiliations:** 1 College of Medicine, King Saud bin Abdulaziz University for Health Sciences, Riyadh, Saudi Arabia; 2 King Abdullah International Medical Research Center, Riyadh, Saudi Arabia; 3 College of Sciences and Health Professions, King Saud bin Abdulaziz University for Health Sciences, Riyadh, Saudi Arabia; 4 College of Medicine, Alfarabi Colleges, Riyadh, Saudi Arabia; Ohio State University Wexner Medical Center, UNITED STATES

## Abstract

**Objectives:**

*KRAS*, *NRAS*, and *BRAF* mutations are commonly present in colorectal cancer (CRC). We estimated the frequency of *KRAS*, *NRAS*, and *BRAF* mutations and assessed their impact on survival and other clinical variables among Saudi patients.

**Design:**

Retrospective cohort study design.

**Settings:**

Oncology department of a tertiary hospital in Riyadh, Saudi Arabia. We gathered information from 2016 to 2018.

**Participants:**

Cohort of 248 CRC patients to assess the demographic data, pathological tumour features, response to treatment modalities, disease progression, and metastasis.

**Statistical analysis used:**

Correlation analysis using the chi-square test. Survival analysis using a Kaplan Meier method. Cox regression analysis to calculate the hazard ratios.

**Results:**

Demographic data revealed that 84% of patients were diagnosed with CRC above the age of 50 years. Only 27% of patients presented with distant metastasis. *KRAS* mutations were the most prevalent (49.6%), followed by *NRAS* mutations (2%) and *BRAF* mutations (0.4%). Wild type tumours were found among 44.4% of patients. *KRAS* mutation showed no significant correlation with the site, type, pathological grade, and stage of the tumour.

The mean survival time was shorter among patients with *KRAS* mutations than among patients with wild type *KRAS* tumours (54.46 vs. 58.02 months). Adjusted analysis showed that the survival time was significantly affected by patients’ age at diagnosis (P = 0.04). Male patients had an increased risk of mortality by 77% (hazard ratio: 1.77).

**Conclusions:**

Saudi CRC patients had a high frequency of *KRAS* mutations and a low frequency of *BRAF* mutations. The *KRAS* mutation status did not affect the patients’ survival.

## Introduction

According to the Saudi Cancer Registry, colorectal cancer (CRC) is the most commonly diagnosed cancer among males and the third commonest among females [1]. Despite advancements made in CRC diagnosis and multimodal treatment, it remains the third commonest cause of cancer-related deaths worldwide; accounting for over half a million deaths annually [2,3]. CRC is a heterogeneous disease that arises from multiple cellular and genetic alterations. This can affect diagnosis and treatment. Efforts aimed at identifying molecular phenotypes for CRC are critical to provide more accurate prognoses, predict tumour response to treatment, and guide targeted therapies [2,4].

Advanced characterisation of the genetic alterations in CRC has suggested that the epidermal growth factor receptor (EGFR) and the downstream RAS–RAF–BRAF–MAPK pathways play a significant role in disease development [5]. *RAS* is a family of genes that encode for guanosine triphosphatases. The first identified *RAS* genes were *HRAS*, *KRAS*, and *NRAS*. RAS can subsequently activate other proteins that regulate transcription factors involved in cell cycle progression, apoptosis, differentiation, and proliferation [6].

*KRAS* mutations are found in approximately 30–50% of CRC lesions and comprise activating point mutations mostly in codons 12 and 13 and rarely in codon 61 [7,8]. Since KRAS is an important downstream component of the EGFR pathway, mutations in the coding gene result in a constitutively activated signalling cascade that acts independently from upstream signals. Prior studies of EGFR inhibitors (cetuximab and panitumumab) in metastatic CRC (mCRC) showed controversial results regarding therapeutic outcomes [9,10]. Responses to treatment were observed only in individuals with wild type KRAS tumours. Thus, KRAS mutations are now recognised as predictors of resistance to EGFR inhibitors [6]. However, the effect of KRAS on prognosis is still debatable, despite several investigations. Many studies have advocated that KRAS is a negative prognostic marker, while others have reported no prognostic significance [6,11].

Even among patients with wild type KRAS CRCs, responses to EGFR inhibitors were not observed among all cases, suggesting that other mutations downstream to EGFR might also lead to a lack of response to treatment [5]. *NRAS* mutations occur in approximately 3–5% of CRCs and are usually found in codon 60 [4]. Since *NRAS* mutations are rare and mainly investigated for their effect on resistance to therapy, their role as prognostic markers is not conclusively reported [4,12].

RAF is a family of serine/threonine kinases that function as direct downstream targets of RAS and is composed of ARAF, BRAF, and CRAF. *BRAF* mutations are identified in approximately 5–20% of CRC lesions and are usually located at codon 600 [4]. Mostly, *KRAS* and *BRAF* mutations are mutually exclusive molecular events. A recent study conducted in Korea reported that Asian populations (including the Saudi Arabian population) had a lower incidence of *BRAF* mutations in mCRC [11]. However, present data are still insufficient to support this hypothesis. As with *RAS* mutations, *BRAF* mutations result in hyper-activation of the MAPK pathway. Therefore, *BRAF* mutations can be considered as a source of resistance to EGFR inhibitors [5]. However, current evidence is inadequate to conclusively evaluate their role as predictive markers [4,11].

In the past few years, considerable advances have been made in the elucidation of molecular alterations underlying mCRC. However, no definitive conclusions have been drawn regarding the role of *KRAS*, *BRAF*, and *NRAS* mutations as predictive and prognostic markers [2,11]. The present study aimed to estimate the prevalence of *KRAS*, *NRAS*, and *BRAF* mutations and highlight their prognostic value among Saudi patients. This can assist in offering a more accurate prognosis and personalising therapeutic approaches based on the presence or absence of biomarkers.

## Methods

We conducted this retrospective cohort study at the oncology department of a tertiary hospital in Riyadh, Saudi Arabia. Since genetic testing started in 2016, we retrospectively reviewed all medical records of CRC patients who were admitted to the hospital between 2016 and 2018 and underwent surgery or were treated with chemotherapy, radiotherapy, or both (n = 248). There were no exclusion criteria. We used a non-probability consecutive sampling technique for all patients who met the inclusion criteria.

The research group members, using patients’ medical records from the Best Care system, collected the data from September 2018 to January 2019. Patients’ records were then reviewed until the 1^st^ of January 2021 to estimate the survival time. We collected data regarding variables such as demographic characteristics (age and gender) and pathological tumour features (type, primary site, grade of differentiation, and staging). The overall mean survival time was defined as the mean of an interval between the date of diagnosis and death or last follow up.

### DNA isolation and analysis of KRAS, BRAF, and NRAS mutations

For DNA isolation, 5–10 μm-thick sections from the patient formalin fixed paraffin-embedded tissue samples were used. Neoplastic cells then were lysed to extract genomic DNA and perform Realtime PCR amplification. Biocartis Idylla^Tm^ KRAS mutations test was done to detect the presence of 21 *KRAS* mutations in exons 2,3, and 4. Biocartis Idylla^Tm^ NRAS-BRAF mutations test was also performed to detect the presence of 18 mutations in *NRAS* exons 2,3, and 4 as well as 5 mutations in *BRAF* exon 15. The tumour mutational status and type of mutation were reported in the molecular testing reports provided by the pathology centre of our hospital.

### Patient and public involvement

The study design was approved by King Abdullah’s International Medical Research Centre ethics review board. Patients and public were not involved in any stage of the research process. The need for informed consent was waived on account of the retrospective nature of the study. Confidentiality and anonymity were maintained throughout the study. No names or medical record numbers was taken from patients’ records. Only the research team had access to the data during the study and after completion. All data were retrieved from the Best Care system of King Abdulaziz Medical City in Riyadh from September 2018 to January 2019, and all were anonymized before accessing them. Patients sought treatment at different times between 2016 to 2018 depending on date of diagnosis.

### Statistical analyses

We used frequencies and percentages to describe categorical data such as gender and mutation status. We described numerical data as the mean and standard deviation or the median and interquartile range, as appropriate. The frequencies of *KRAS*, *NRAS*, and *BRAF* mutations were estimated at a 95% confidence level. Due to the low number of patients with *BRAF* and *NRAS* mutated CRCs, these mutations were excluded from the survival analysis. We used the Chi-Squared test to assess the association between *KRAS* mutations and other clinical variables. A Kaplan Meier analysis was done to compare the mean survival time between patients with *KRAS* mutated and wild type CRCs. Hazard Ratios (HR) were calculated by the Cox regression analysis stratified by patients’ gender and age, tumour stage and mutational status. All statistical analyses were performed using SPSS version 22 (IBM Corporation, Armonk, NY, USA). Analysis items with a P-value of <0.05 were considered statistically significant.

## Results

The present analysis included 248 CRC patients. Males represented a majority of patients compared to females (62% vs. 38%). The mean age at diagnosis was 63 ± 14 years with 84% of patients diagnosed above the age of 50 years. Approximately 45% of CRC patients had complete or partial response to treatment, and 27% had disease progression or metastasis.

The commonest primary tumour site was the rectum (27%), followed by the right colon (25%). Anorectal tumours were the least common and accounted for 0.4% of cases. Histologically, 99% of CRCs were adenocarcinomas in origin and 83% were moderately differentiated tumours. Advanced tumour stage (T stage) and metastasis stage (M stage) at the initial diagnosis had a negative impact on patients’ prognoses (P<0.001). Stage T3 (50%) and stage N1 (36%) were the most commonly reported stages. A large proportion of patients presented with stage M1 disease (28.2%).

**Fig 1:**
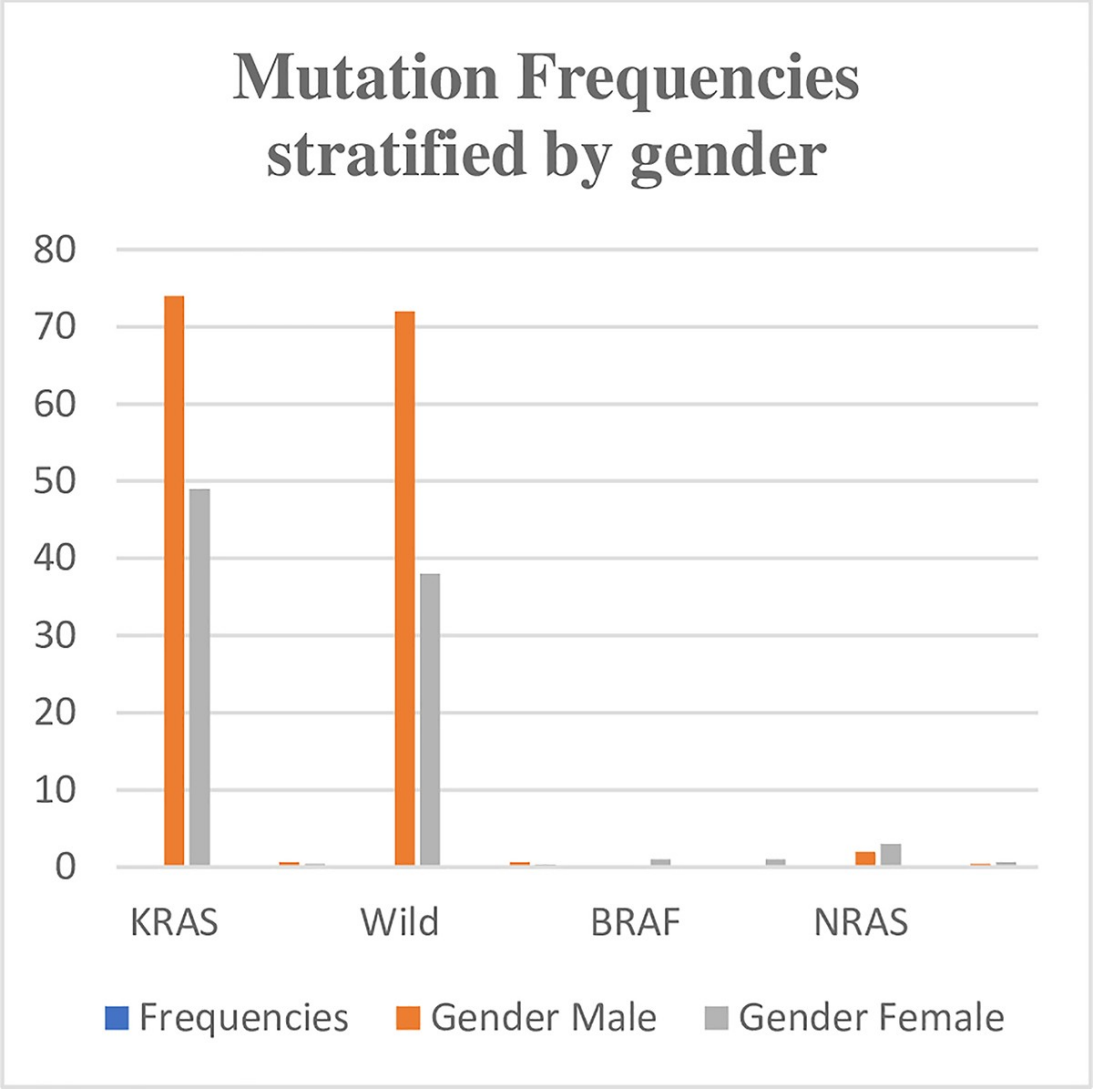
Distribution of mutation frequency of *KRAS*, *NRAS* and *BRAF* genes among 248 CRC patients stratified by gender.

**Fig 2:**
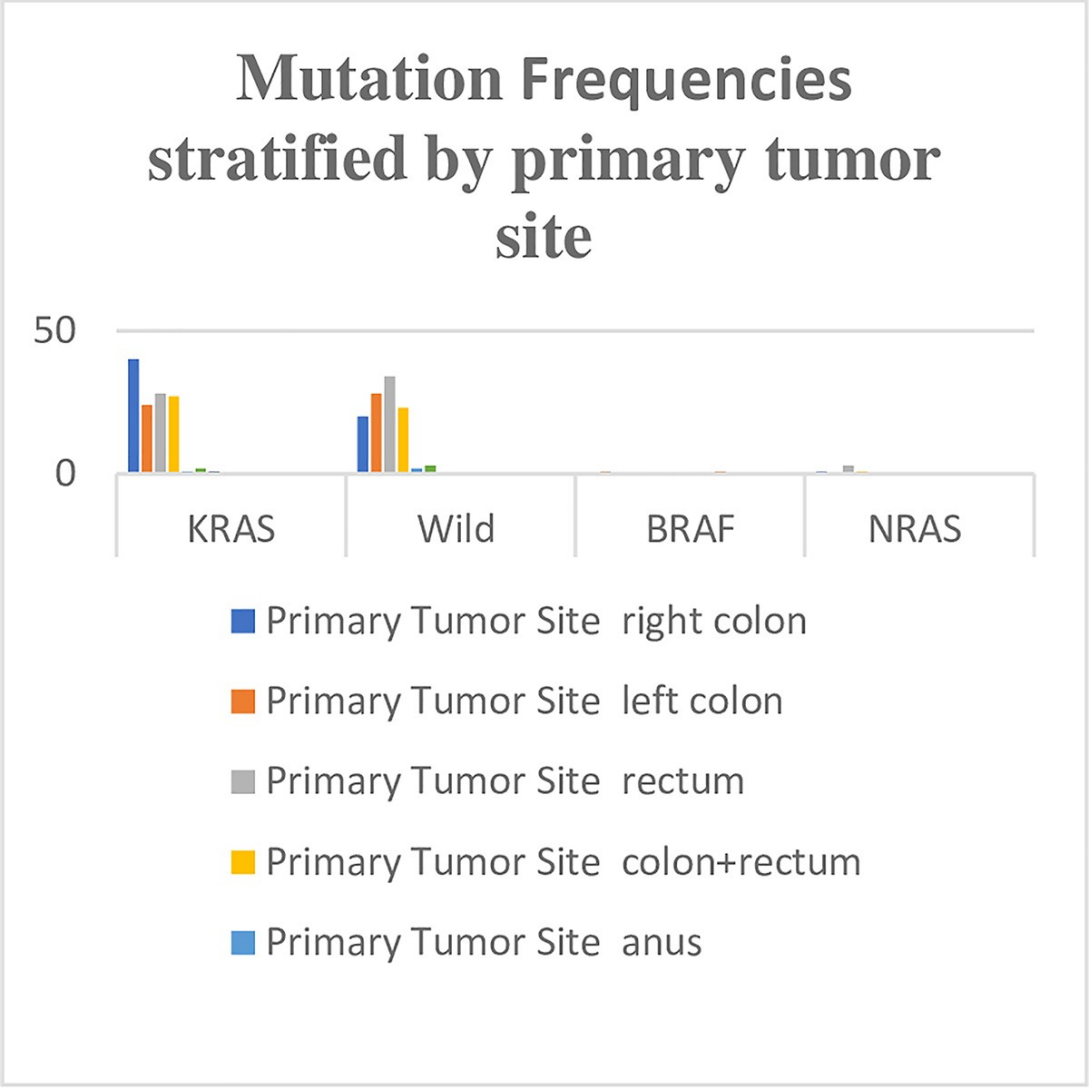
Distribution of mutation frequency of *KRAS*, *NRAS* and *BRAF* genes among 248 CRC patients stratified by primary tumor site.

*KRAS* mutations were most frequently detected among CRC patients (49.6%), followed by *NRAS* mutations (2%) and *BRAF* mutations (0.4%). Wild type CRCs were detected among 44.4% of patients (Figs 1 and 2). Compared to wild type *KRAS* CRCs, *KRAS* mutated tumours showed a trend toward female patients (56.3% vs. 43.7%), patients with right-sided tumours (66.7% vs. 33.3%), and a moderately differentiated histology (53.6% vs. 46.4%). However, these differences were not statistically significant (P = 0.404; P = 0.147; P = 0.436) (Table 1).

**Table 1:** Results of Chi-Squared tests of association between mutational status and demographic and clinicopathological characteristics.

Variable	Category	Mutation status		P-value
		*KRAS*	Wild type	
		n (%)	n (%)	
**Gender**	Male	74 (50.7%)	72 (49.3%)	0.404
	Female	49 (56.3%)	38 (43.7%)	
**Age, years**	<50	18 (46.2%)	21 (53.8%)	0.455
	51–60	35 (54.7%)	29 (45.3%)	
	61–70	35 (60.3%)	23 (39.7%)	
	≥71	35 (48.6%)	37 (51.4%)	
**T stage**	T1	1 (33.3%)	2 (66.7%)	0.725
	T2	14 (45.2%)	17 (54.8%)	
	T3	64 (53.3%)	56 (46.7%)	
	T4	28 (52.8%)	25 (47.2%)	
	Undocumented	16 (61.5%)	10 (38.5%)	
**N stage**	N0	37 (54.4%)	31 (45.6%)	0.533
	N1	46 (52.9%)	41 (47.1%)	
	N2	21 (44.7%)	26 (55.3%)	
	X	1 (33.3%)	2 (66.7%)	
	Undocumented	18 (64.3%)	10 (35.7%)	
**M stage**	M0	29 (43.9%)	37 (56.1%)	0.092
	M1	35 (53.8%)	30 (46.2%)	
	MX	42 (64.6%)	23 (35.4%)	
	Undocumented	17 (45.9%)	20 (54.1%)	
**Location**	Right colon	40 (66.7%)	20 (33.3%)	0.147
	Left colon	24 (46.2%)	28 (53.8%)	
	Rectum	28 (45.2%)	34 (54.8%)	
	Colon and rectum	27 (54%)	23 (46%)	
	Anus	1 (33.3%)	2 (66.7%)	
	Small intestine	2 (40%)	3 (60%)	
	Anorectal	1 (100%)	0 (0%)	
**Prognosis**	Response to treatment	53 (49.5%)	54 (50.5%)	0.509
	No response to treatment	33 (52.4%)	30 (47.6%)	

**Fig 3:**
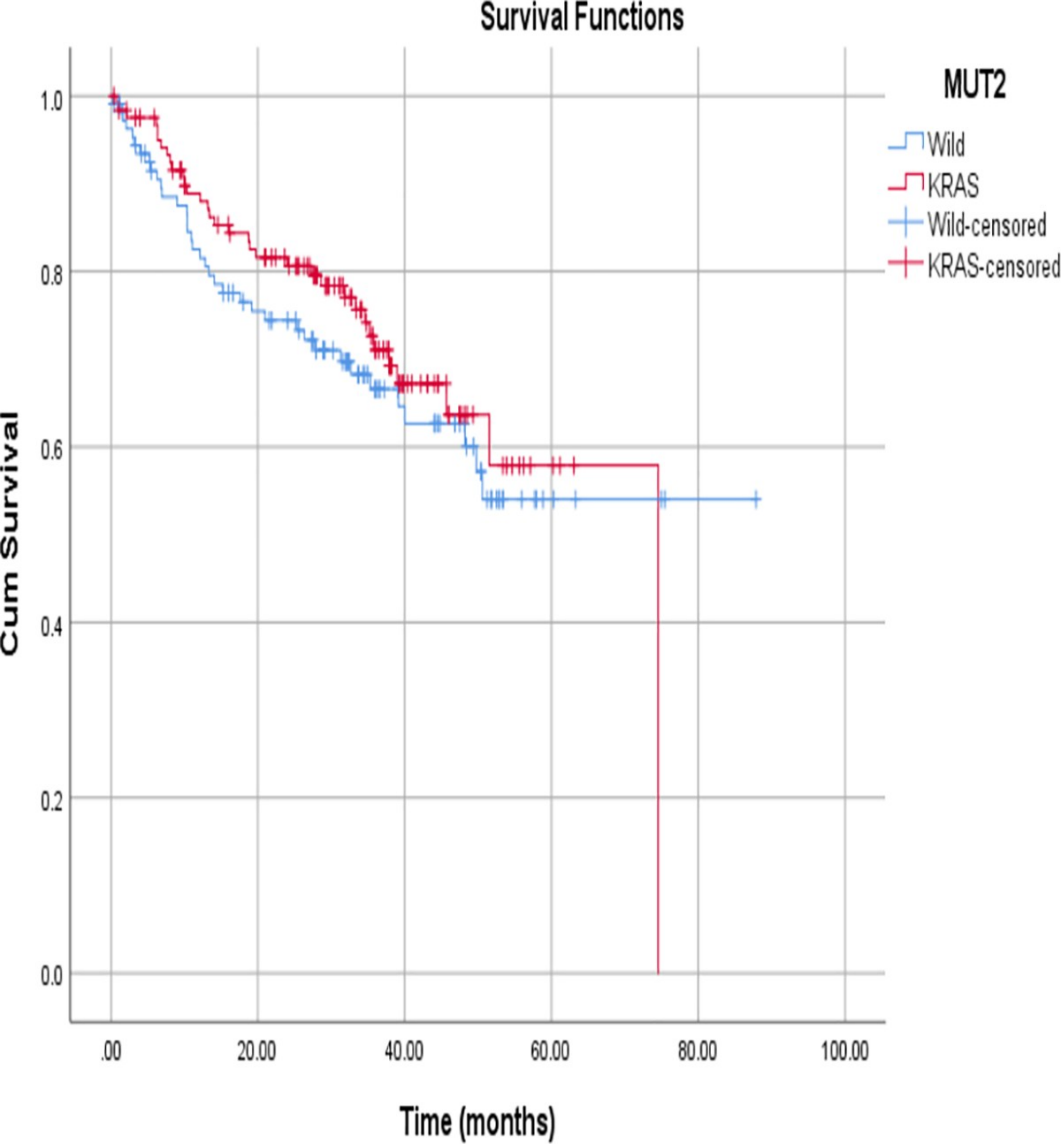
A Kaplan Meier analysis of KRAS mutated tumours vs wild-type tumours.

The mortality rate was estimated to be 31.9%. The overall mean survival time was 58.22 months. The mean survival time was shorter among patients with *KRAS* mutations than among patients with wild type *KRAS* tumours (54.46 vs. 58.02 months), but the difference was not statistically significant (P = 0.444) (Fig 3). The survival time was better among females compared to males (62 vs. 50.75 months), but this was not statistically significant (P = 0.518).

Adjusted analysis showed that the survival time was significantly affected by patients’ age at diagnosis (P = 0.04). Patients who are younger than 60 years had a lower risk of mortality. Compared to those aged under 50 years or over 60 years, patients aged between 51–60 years had the lowest risk of mortality. Moreover, presence of metastasis significantly increased the risk of death (hazard ratio: 3.81, P = <0.001). The difference between males and females for the hazard of mortality approached the borderline of significance (*P* = 0.08). Male patients had an increased risk of mortality by 77% (hazard ratio: 1.77). There was no significant difference between *KRAS* mutated tumours and wild type *KRAS* tumours for the hazard of mortality (hazard ratio:1.68, P = 0.09) (Table 2).

**Table 2:** Results of Cox regression analysis of the hazard ratios stratified patients’ gender and age, tumour stage and mutational status.

Variable	Category	P-value	HR	95% CI for HR	
				Lower	Upper
**Gender**	Male	0.081	1.77	0.93	3.36
	Female*		1.00		
**Age**	= < 50	0.042	0.41	0.18	0.97
	51–60	0.002	0.26	0.11	0.61
	61–70	0.091	0.54	0.26	1.10
	= 71+ *		1.00		
**Stage T**	T2	0.851	1.09	0.44	2.70
	T3	0.238	0.68	0.35	1.30
	T4*		1.00		
**Stage N**	N0	0.839	0.91	0.369	2.248
	N1	0.417	0.737	0.352	1.541
	N2*		1.00		
**Stage M**	M0	0.256	0.61	0.26	1.44
	M1	0	3.81	1.83	7.93
	Mx*		1.00		
**Mutation**	Wild	0.099	1.68	0.91	3.11
	KRAS*		1.00		

## Discussion

*KRAS* mutations were found in 49% of CRCs. This proportion was higher than those reported in western countries and the Asian region (35–40%) [13,14]. However, it is in accordance with the mutation rates reported in Iraq, Spain, and Slovenia (46.2–48.0%) [15,16]. On the contrary, *BRAF* mutations were observed in only 0.4% of CRCs. To the best of our knowledge, this is the lowest proportion reported in the literature. The mutation rates reported in western countries ranged from 10–15% and are estimated to be 4% in the Asian region. *NRAS* mutations were evident in 2% of CRCs, which is similar to global estimates (3–5%) [13,14].

In this study, the age of the patient did not significantly affect the mutation status. Instead, CRCs among female patients were likelier to harbour *KRAS* mutations. Approximately 56% of female and 50% of male patients had *KRAS* mutations. This finding corresponds to the results of other studies conducted in the US, Indonesia, and China [14,17,18]. The reasons behind this phenomenon are not yet known. We found no significant difference in the tumour stage at diagnosis between mutant and wild type CRCs. Moreover, the type of tumour and grade of differentiation was unaffected by the mutation status. Both findings are in accordance with those of similar studies [14,19]. Right colon tumours were likelier to harbour *KRAS* mutations than left colon tumours; similar findings were observed in previous studies [18,19]. This could explain the relationship between right colon tumours and poor prognosis.

The survival times among patients with *KRAS* mutant and wild type CRCs were estimated to be 54 months and 58 months, respectively. Although this difference is not statistically significant, it is clinically significant.

The survival rate among CRC patients was estimated to be 68%. According to the Saudi Cancer Registry, the 5-year survival rate among CRC patients from 1994 to 1999 was 44% [20]. We believe that this significant difference is linked to an improvement in diagnostic and treatment modalities. Moreover, our data were extracted from a tertiary hospital with better patient care and multidisciplinary teams. By contrast, the Saudi Cancer Registry included all CRC patients from different cities in multiple facilities.

CRC was more prevalent among male patients (61.7%) than among female patients (38.3%), consistent with the results of other studies conducted worldwide [2,3]. According to a study published in the *Annals of Saudi Medicine* in 2015, Saudi males had higher rates of CRC than did males in both developed and developing countries, while females had similar rates to those detected in developed countries [21]. This disparity between the sexes is not well understood; however, it could be linked to increased exposure to risk factors such as smoking and red meat consumption [22].

The median age at diagnosis of colon cancer in the United States (US) is 68 years among males and 72 years among females [20]. In the present study, the median ages for the development of CRC were 63 years and 61 years among males and females, respectively. The younger median age at which Saudi patients are diagnosed compared to patients in the US is possibly linked to a younger Saudi population. It is estimated that 71.1% of the Saudi population is younger than 45 years [20]. This observation highlights the importance of screening Saudi patients earlier than at 50 years, which is considered the standard age for CRC screening in the US.

In the present study, the proportion of Saudi patients presenting with distant metastases was approximately 27%, which corresponds to other results reported locally. According to the Saudi Cancer Registry, the percentage of patient presented with distant metastasis was 29.2% and 28.4% in 2008 and in 2010 respectively [20]. In the US, about 20% of patients have tumour metastasis. Moreover, the proportion of patients presenting with localised CRCs in the present study was approximately 15%, compared to 39% in the US [17]. This indicates that proportionally more Saudi patients present at an advanced stage. This may be a result of CRC screening programmes in the US, which are lacking in Saudi Arabia.

### Limitations

The genetic testing started three years ago in our hospital, which affected the total number of patients with known mutation status. Instead of 447 patients, which was the recommended sample size, only 248 patients were included in this study. Moreover, this study was conducted in a single tertiary hospital. Such limitations might affect both the overall distribution of variables, and the identified associations as well as the generalizability of the study results. Since only one patient had *BRAF* mutation, and five patients had *NRAS* mutation, this study could not assess the impact of *BRAF* and *NRAS* mutations on patients’ survival.

## Conclusions

Saudi patients had a higher frequency of *KRAS* mutations and a lower frequency of *BRAF* mutations when compared to Asian and western countries. *KRAS* mutation status did not impact CRC patients’ survival. Further local studies with a larger number of patients are necessary to determine the frequency of *KRAS*, *NRAS* and *BRAF* mutations and to truly understand their impact on prognosis and survival.
